# Assessing the Efficacy of an AI-Powered Chatbot (ChatGPT) in Providing Information on Orthopedic Surgeries: A Comparative Study With Expert Opinion

**DOI:** 10.7759/cureus.63287

**Published:** 2024-06-27

**Authors:** Andrew M Smith, Evan A Jacquez, Evan H Argintar

**Affiliations:** 1 Orthopedic Surgery, Georgetown University School of Medicine, Washington DC, USA; 2 Orthopedic Surgery, MedStar Georgetown University Hospital, Washington DC, USA; 3 Orthopedic Surgery, MedStar Washington Hospital Center, Washington DC, USA

**Keywords:** machine learning, patient education, clinical orthopaedics, ai, chatgpt

## Abstract

Background

The use of artificial intelligence (AI) as a tool for patient care has continued to rapidly expand. The technology has proven its utility in various applications across several specialties in a variety of applications. However, its practicality in orthopedics remains widely unknown. This study seeks to determine if the open-access software Chat Generative Pre-Trained Transformer (ChatGPT) can be a reliable source of data for patients.

Questions/purposes

This study aims to determine: (1) Is the open-access AI software ChatGPT capable of accurately answering commonly posed patient questions? (2) Will there be a significant difference in agreement among the study experts in the answers generated by ChatGPT?

Methods

A standard list of questions for six different procedures across six subspecialties is posed to ChatGPT. The procedures chosen were anterior cruciate ligament (ACL) reconstruction, microdiscectomy, total hip arthroplasty (THA), rotator cuff repair, carpal tunnel release, and ankle fracture open reduction and internal fixation. The generated answers are then compared to expert opinion using a Likert scale based on the agreement of the aforementioned experts.

Results

On a three-point Likert scale with 1 being disagree and 3 being agree, the mean score across all subspecialties is 2.43, indicating at least partial agreement with expert opinion. There was no significant difference in the Likert scale mean across the six subspecialties surveyed (p = 0.177).

Conclusions

This study shows promise in using ChatGPT as an aid in answering patient questions regarding their surgical procedures. This opens doors for the use of the software by patients for understanding and increased shared decision-making with their surgeons. However, studies with larger participation groups are necessary to ensure accuracy on a larger and broader scale as well as studies involving specific application of AI within surgeon's practice.

## Introduction

The integration of artificial intelligence (AI) across various industries has sparked a paradigm shift, revolutionizing traditional processes and unlocking new possibilities. In the realm of healthcare, AI stands out as a powerful tool with the potential to significantly improve patient care, enhance diagnostic accuracy, and refine treatment strategies. Previous studies in dermatology, radiology, and other specialties have highlighted its potential as a diagnostic and educational tool, even outperforming physicians in some cases [1-3]. The transformative technology has gradually permeated different facets of healthcare delivery, offering innovative solutions to longstanding challenges [4].

Within the domain of orthopedic surgery, AI's integration represents a particularly promising avenue for driving positive change. Orthopedic procedures, ranging from joint replacements to spinal surgeries, are often intricate and require meticulous planning and execution. Moreover, patients undergoing such surgeries commonly have a multitude of questions regarding the procedure itself, the recovery process, and the expected outcomes and often feel as though they do not receive all of the information they need [5]. In this context, the provision of clear and accurate information is paramount, facilitating informed decision-making and fostering patient satisfaction.

AI technologies, exemplified by the Chat Generative Pre-Trained Transformer (ChatGPT) in this study, present a compelling solution to address the informational needs of patients considering orthopedic surgeries [6]. Through natural language processing capabilities, ChatGPT can engage with patients in conversational exchanges, elucidating complex concepts and addressing queries in a manner that is accessible and easily comprehensible to many [7]. Given patients often turn to the internet and resources other than their physicians for information, web-based AI software poses a unique solution [8]. By leveraging AI in this capacity, healthcare providers have the opportunity to enhance patient education, communication, and overall surgical experiences, thereby bolstering patient outcomes and satisfaction.

The primary objective of this study was to evaluate ChatGPT's efficacy in responding to common questions pertaining to orthopedic surgeries, as identified by expert orthopedic surgeons. Previous studies have evaluated ChatGPT’s orthopedic knowledge based on that of a first-year post-graduate resident physician [9]. We aim to gain deeper insights into the strengths and limitations of AI in patient education within the orthopedic surgery domain. Through this investigation, we seek to bridge the gap between medical expertise and patient information, elucidating the transformative potential of AI in augmenting patient experiences and outcomes in orthopedic surgery.

## Materials and methods

The study was a qualitative surgery design at a major academic center in a large urban setting. Experts in subspecialties were recruited and data was collected over a period of weeks, differing by surgeon response rate. The data collected was obtained from subjects deemed as experts in their respective subspecialties of orthopedics. To be included as an expert opinion, the surgeon must have been board-certified in the subspecialty within which they were addressing their responses. Length of independent practice, although acknowledged as important, was not used for exclusion.

To begin gathering data, a comprehensive list of 10 common questions that patients commonly ask about orthopedic surgeries was compiled (Table 1). To assess ChatGPT’s overall effectiveness, these questions were devised using input from expert orthopedic surgeons for six of the most common orthopedic surgeries, anterior cruciate ligament (ACL) reconstruction, microdiscectomy, total hip arthroplasty (THA), carpal tunnel release, rotator cuff repair, and ankle open reduction internal fixation (ORIF), across six subspecialties. Rupture of the ACL of the knee is an unfortunate but common occurrence in the athletic population, with reconstruction consisting of arthroscopically-assisted placement of a tendon graft into the knee joint to function as the ACL and re-stabilize the knee joint. Minimally invasive microdiscectomy is performed for symptomatic intervertebral disc herniations (most commonly in the lumbar spine) and consists of the removal of disc material from the spinal canal to liberate compressed nerves with maximal preservation of facet joints and posterior elements. THA involves the replacement of the articulating surfaces of the hip joint, the acetabulum, and the femoral head, with artificial components to improve mobility and reduce or eliminate pain in patients with osteoarthritis or other degenerative conditions affecting the joint. Carpal tunnel release is indicated for patients with peripheral compression of the median nerve, causing pain, paresthesias, and even weakness at later stages. The transverse carpal ligament is incised, thus freeing the contents of the carpal tunnel from superficial compression. Rotator cuff tears can occur in a variety of settings and severities, causing pain and weakness with shoulder movements. Some such tears are amenable to direct repair, most commonly performed arthroscopically with suture anchors re-approximating tendon(s) to the head of the humerus. Finally, ankle injuries are a common cause of emergency department visits, frequently with radiographs revealing a fracture of either the tibia or fibula (or both) at the level of the ankle joint. Should these be deemed operative, surgical fixation is typically performed with plate and screw fixation in an open fashion.

**Table 1:** Curated question list to be posed to ChatGPT.

	ChatGPT question list
1.	Why do I need this surgery?
2.	Are there alternatives to surgery?
3.	How is the surgery performed?
4.	Will this surgery solve the problem?
5.	What will happen if I don't have surgery?
6.	What are the risks of the surgery?
7.	Will I be in pain after the surgery?
8.	How long will the recovery be?
9.	Will I be able to do all the activities I did before?
10.	Will I need any more surgeries after?

Before the questions were proposed to ChatGPT, the model was tuned to ensure accurate and contextually relevant responses. The AI was prompted to prepare to answer questions regarding orthopedic surgeries as if they were being asked by a patient. A panel of experienced orthopedic surgeons was assembled to assess the accuracy of the answers to the same set of questions. The expert panel was an intentionally diverse grouping, with representatives from each subspecialty within orthopedic surgery. The surgeons were then asked to evaluate the accuracy, comprehensiveness, and clarity of responses from ChatGPT. Their responses were recorded as either fully agree, partially agree, or disagree with the ChatGPT-generated answers.

Using a Likert scale, expert opinions reported as fully agree, partially agree, or disagree were transformed into quantitative data assigning 3 as fully agree, 2 as partially agree, and 1 as disagree. The entirety of the orthopedic surgery department was contacted at a major academic center to obtain the largest number of responses possible. Additional surveys were presented to affiliates of the department around the country. The data was compiled using the aforementioned Likert scale and SPSS was used to obtain the mean, SD, and 95% confidence interval for the data obtained. An ANOVA analysis was performed with the converted data to determine if there was a significant difference amongst the responses by subspecialty with a significance level set at a p-value of <0.05.

## Results

The findings of this study underscore ChatGPT's remarkable capability to provide precise and insightful responses to simulated patient questions, aligning closely with an expert opinion within the field of orthopedic surgery. Through our evaluation process, ChatGPT demonstrated its proficiency in synthesizing answers that resonated with the insights of orthopedic surgeons surveyed across various subspecialties.

When confronted with inquiries spanning a spectrum of orthopedic procedures, including ACL repair, microdiscectomy, THA, carpal tunnel release, rotator cuff repair, and ankle ORIF, ChatGPT consistently delivered responses that garnered favorable ratings on the Likert scale. With an average score of 2.43 ± 0.59 (n=23), ChatGPT's responses received more than partial agreement across expert evaluators, indicative of its ability to provide accurate and reliable information in line with expert surgical knowledge (Table 2).

**Table 2:** Resultant descriptive statistics for applied Likert Scale. Likert Scale Applied: 1: disagree; 2: partial agreement; 3: full agreement N: number of expert surgeons; CI: 95% confidence intervals for means; Std. deviation: standard deviation; Std. error: standard error; ORIF: open reduction internal fixation; ACL: anterior cruciate ligament

	N	Mean	Std. deviation	Std. error	Lower CI	Upper CI	Minimum	Maximum
ACL repair	4	2.25	0.5	0.25	1.45	3.05	2	3
Microdiscectomy	4	3	0	0	3	3	3	3
Total hip arthroplasty	4	2.25	0.5	0.25	1.45	3.05	2	3
Carpal tunnel release	5	2.2	0.837	0.374	1.16	3.24	1	3
Rotator cuff repair	4	2.25	0.5	0.25	1.45	3.05	2	3
Ankle ORIF	2	3	0	0	3	3	3	3
Total	23	2.43	0.59	0.123	2.18	2.69	1	3

Analyzing the individual means for each procedure further highlights ChatGPT's proficiency in addressing a diverse range of orthopedic inquiries. Across all procedures, ChatGPT's responses garnered favorable ratings, with individual means being 2.25 ± 0.5, 3 ± 0, 2.25 ± 0.5, 2.20 ± 0.837, 2.25 ± 0.5, and 3.00 ± 0, respectively, for ACL repair, microdiscectomy, THA, carpal tunnel release, rotator cuff repair, and ankle ORIF. Furthermore, there was no significant disparity in agreement among experts regarding the quality of ChatGPT's responses across different subspecialties, as evidenced by the non-significant p-value of 0.177 obtained from the ANOVA analysis of descriptive means (Table 3).

**Table 3:** ANOVA analysis of means taken from descriptive data. ANOVA: analysis of variance

	Sum of squares	df	Mean square	F	P-value
Between groups	2.602	5	0.52	1.752	0.177
Within groups	5.05	17	0.297		
Total	7.652	22			

These findings underscore the robustness and consistency of ChatGPT's performance in providing accurate and informative responses across various orthopedic procedures. By aligning closely with expert opinion and eliciting agreement from expert evaluators, ChatGPT demonstrates its potential as a valuable tool for augmenting patient education and communication within the field of orthopedic surgery. Moving forward, continued refinement and validation of AI-powered solutions like ChatGPT hold promise for enhancing patient experiences and outcomes in orthopedic care, while complementing the expertise of human clinicians.

## Discussion

Our study has shown that the web-based AI engine ChatGPT is capable of accurately answering common patient questions pertaining to surgical procedures, as verified by agreement with expert opinion. These findings are consistent with previous studies that have also shown the usefulness of open AI software in their ability to provide information on orthopedic procedures in arthroplasty and hand [10-12]. These studies concluded that ChatGPT provided responses that coincided with evidence and presented it in a manner that would be useful to patients. They showed that the majority of questions generated by the AI software needed minimal to moderate clarification, while also highlighting the engine's repeated expression that the results be discussed with a surgeon, thus appropriately advising the user to seek the necessary clarification [10-12].

Our study also set out to determine if ChatGPT could respond to common patient questions across multiple subspecialties in a reliable manner. We found that the openly available AI software produced responses that did not significantly differ in their agreement across six different procedures/subspecialties. These findings demonstrate the depth and range of information ChatGPT is able to generate as well as its applicability to broad subject matter. The results also showcase the ability of ChatGPT to provide accurate answers to questions with a spectrum of complexity. These findings open the door for the potential application of ChatGPT as a reliable resource for surgeons to provide to their patients. These findings build on previous trials that illustrated the ability of AI to be used as an educational implement for patients while obtaining informed consent [13]. It also aligns with literature showing its ability to improve the health literacy of patients and the general readability of medical literature [7,14].

This study has a number of limitations. The first limitation is the relatively small number of responses by expert orthopedic surgeons. With a total participation of 23 across 6 different procedures, the average number of responses per procedure was just under 4. Furthermore, it is important to acknowledge differences in the opinion of surgeons and their preferred treatment courses whenever using them as the basis for comparison. However, all surgeons surveyed are board-certified in subspecialties related to the procedures in question. The ChatGPT answers were also provided by the engine without source material as a reference, providing a challenge but also highlighting the necessity of this study and of shared decision-making with a surgeon [15].

## Conclusions

This study revealed that many orthopedic surgeons recognize the value of AI, represented in this instance by ChatGPT, in patient education and communication. They perceive AI as a supportive resource that enhances patient engagement, streamlines healthcare delivery, and empowers patients in their healthcare decisions. It also establishes the potential for AI to be used broadly, across subspecialties in orthopedics. The authors would like to state that while AI's role in orthopedic surgery is viewed positively by these surgeons, it is important to acknowledge that AI should complement, not replace, the expertise and empathy of healthcare providers. Future studies and implementations should aim to strike the right balance between AI and human interactions to maximize the benefits for both patients and healthcare professionals.
